# Monoclonal stool antigen test for diagnosing *Helicobacter pylori* in chronic atrophic gastritis: a prospective primary care study

**DOI:** 10.3389/fcimb.2026.1811628

**Published:** 2026-04-13

**Authors:** Cheng Fang, Peiwei Li, Dingjiacheng Jia, Yan Li, Songzhao Zhang, Rong Zhang, Lin Lu, Yuehua Han

**Affiliations:** 1 Department of Gastroenterology, the Second Affiliated Hospital of Zhejiang University School of Medicine, Hangzhou, Zhejiang, China; 2 Department of Clinical Laboratory, the Second Affiliated Hospital of Zhejiang University School of Medicine, Hangzhou, Zhejiang, China; 3 Department of Medical Administration, Hangzhou Women ‘s Hospital, Hangzhou, Zhejiang, China

**Keywords:** atrophic gastritis, diagnostic accuracy, *Helicobacter pylori*, monoclonal stool antigen test, risk stratification

## Abstract

**Background/aims:**

Diagnosing active *Helicobacter pylori* (*H. pylori*) infection in patients with chronic atrophic gastritis (CAG) remains challenging. The monoclonal stool antigen test (SAT) offers a non-invasive alternative to endoscopic methods. This study aimed to evaluate the diagnostic accuracy of SAT in CAG and to analyze its performance in relation to the severity of gastric atrophy.

**Methods:**

In this prospective study, 287 patients with CAG underwent SAT, urea breath test, serology, and histology. Patients were stratified into low-risk and high-risk groups based on gastric cancer progression risk. SAT was assessed against a reference standard combining histology and UBT.

**Results:**

The area under the ROC curve for SAT was 0.858. SAT showed a sensitivity of 75.9% and a specificity of 96.1%. In high-risk patients, SAT demonstrated comparable specificity (96.77% vs. 95.77%) and positive predictive value (95.24% vs. 93.48%) to those in low-risk patients, with numerically higher accuracy (89.29% vs. 86.05%). Consistency analysis revealed substantial agreement between SAT and the reference standard overall (κ=0.72), with stronger agreement in the high-risk patients (κ=0.77) than in the low-risk patients (κ=0.69).

**Conclusions:**

Monoclonal SAT is a reliable non-invasive tool for detecting *H. pylori* in CAG, exhibiting high specificity and strong diagnostic consistency. Its performance is positively correlated with the severity of gastric atrophy, supporting its preferential use in high-risk, advanced atrophy populations.

## Introduction

Chronic atrophic gastritis (CAG) is a recognized precancerous lesion that significantly increases the risk of gastric cancer, particularly when accompanied by *Helicobacter pylori* (*H. pylori*) infection (Malfertheiner et al., 2022). As a pathogenic bacterium affecting over 50% of the global population, *H. pylori* can induce persistent chronic inflammation and initiate the gastric carcinogenic cascade (Huang et al., 2023). This process may progressively lead to glandular loss, intestinal metaplasia, low-grade adenoma/dysplasia, high-grade adenoma/dysplasia, and ultimately invasive carcinoma in 1-2% of infected individuals. Therefore, accurate detection of active *H. pylori* infection in patients with CAG is of critical clinical importance.

Currently, active *H. pylori* infection can be detected using either invasive methods such as endoscopic biopsy or non-invasive approaches including the urea breath test (UBT) and serological testing. However, it is important to note that the density of *H. pylori* colonization could significantly reduce in CAG, particularly in regions such as the gastric antrum, the lesser curvature of the gastric body, and areas with intestinal metaplasia (IM). This reduction in bacterial load poses specific challenges for biopsy-dependent diagnostic techniques. Although non-invasive tests are less affected by the uneven distribution of bacteria, they also have limitations (Lahner et al., 2004). UBT relies on bacterial urease activity and may yield false-positive results due to urease-producing bacteria other than *H. pylori*, such as those present in the oral microbiota (Chen et al., 2001). Meanwhile, a major drawback of serological testing is its inability to distinguish between active infection and previous exposure, making it more suitable for epidemiological screening rather than confirming active infection (Atkinson and Braden, 2016).

The monoclonal stool antigen test (SAT) has been widely adopted in recent years due to its non-invasiveness, cost-effectiveness, and operational convenience (Gisbert et al., 2006). However, there is a notable scarcity of studies specifically evaluating its diagnostic performance in patients with CAG. In particular, it remains unclear whether the SAT detection threshold is influenced by the degree of gastric atrophy. Therefore, this study aims to evaluate the diagnostic accuracy of the monoclonal SAT for detecting active *H. pylori* infection in patients with CAG.

## Methods

### Patients’ selection

We conducted a prospective comparative study enrolling 287 consecutive patients with CAG, a precancerous condition defined by progressive glandular loss and/or intestinal metaplasia, thinning of the mucosa, chronic inflammatory cell infiltration (lymphocytes, plasma cells) in the lamina propria, and distortion of glandular structure (Zheng et al., 2023). Exclusion criteria included gastrectomy history, active gastrointestinal bleeding, recent (within 4 weeks) use of proton pump inhibitors, H_2_-receptor antagonists, or antibiotics, as well as any prior *H. pylori* eradication therapy. A total of 79 patients meeting one or more exclusion criteria were excluded, yielding 208 eligible participants. All enrolled individuals provided written informed consent and completed standardized medical history questionnaires. Participants were stratified based on atrophy severity and serum gastric function panel results and underwent all diagnostic tests for *H. pylori* infection on the same day as their scheduled health check-up and endoscopy.

The study was conducted in accordance with the Declaration of Helsinki. The study proposals were approved by the Clinical Research Ethics Committee of the Second Affiliated Hospital Zhejiang University School of Medicine (Approval No.Y2021-0290). Written informed consent was provided by all patients before taking part in the study.

### Monoclonal stool antigen test

Fresh stool samples were collected from all participants and analyzed using a commercially available monoclonal antibody-based enzyme immunoassay (EIA) kit (Jingjin Wolf Bioengineering Tech, Beijing). Laboratory personnel were blinded to the *H. pylori* infection status of the patients. The test operates by capturing *H. pylori* antigens in microwells via monoclonal antibodies. This is followed by the addition of a peroxidase-conjugated antibody, incubation, washing, and subsequent colorimetric detection.

### Urea breath test

All participants underwent a urea breath test (UBT) on the same day before endoscopy, using commercially available kits (Huagan Anbang Technologies, Beijing). After an overnight fast, a baseline breath sample was collected. Participants then ingested a test solution containing either ¹³C−urea or ^14^C−urea. A second breath sample was collected 20 minutes after ingestion. The concentration of labeled carbon dioxide in the exhaled breath was measured and compared against the baseline sample measured using the corresponding method. The cutoff values were 4.0 for the ^13^C-urea breath test and 100 dpm/mmol CO^2^ for the ^14^C-urea breath test. A delta over baseline ≥4.0‰ (^13^C−UBT) or a count ≥100 dpm (^14^C−UBT) was considered positive for active *H. pylori* infection.

### Serological examination and gastric function panel

Serum levels of pepsinogen I (PG I), pepsinogen II (PG II), gastrin-17 (G-17), and *H. pylori* immunoglobulin G (IgG) antibodies were measured using commercially available enzyme-linked immunosorbent assay (ELISA) kits, in accordance with the manufacturer’s guidelines (Genelabs, Singapore). As participants with a history of *H. pylori* eradication therapy were excluded based on questionnaire responses, and considering that spontaneous resolution of *H. pylori* infection is uncommon after it is established (NIH Consensus Conference, 1994), a solitary positive serology result may more likely represent a false positive rather than evidence of past infection.

### Histological examination for the detection of *H. pylori* infection and atrophy

All enrolled patients were confirmed to have chronic atrophic gastritis (CAG) based on endoscopic and histopathological findings. Random biopsy samples were collected from five standardized gastric sites following an established biopsy protocol for histopathological evaluation (De Vries et al., 2010). Specifically, two biopsies were obtained from the antrum, two from the angulus, and two from the corpus.

All specimens were assessed by experienced gastrointestinal pathologists. After hematoxylin-eosin and modified Giemsa staining, the specimens were graded in accordance with the updated Sydney classification system. Gastric mucosal atrophy was defined as the loss of native glandular tissue and was quantified using the visual analog scale provided in the updated Sydney classification. The degrees of atrophy and intestinal metaplasia (IM) were each graded as absent (-), mild (+), moderate (++), or marked (+++). Based on the extent of IM, cases were further classified according to the OLGIM (Operative Link on Gastric Intestinal Metaplasia) staging system. OLGIM stages III-IV were defined as high risk for gastric cancer, in combination with the PG I/II ratio.

### Definition of *H. pylori* status

The reference standard for active *H. pylori* infection was predefined prior to data analysis. A patient was classified as true positive only when both the urea breath test (UBT) and histopathological examination yielded positive results. Conversely, a patient was classified as true negative only when both tests were negative. Cases with discordant results (i.e., UBT-positive but histology-negative, or UBT-negative but histology-positive) were considered indeterminate and were excluded from the diagnostic performance analysis. All diagnostic accuracy metrics (sensitivity, specificity, PPV, NPV, and accuracy) for the stool antigen test and serology were calculated against this composite reference standard.

### Statistical analysis

The area under the ROC curve (AUC) with 95% confidence interval (CI) was calculated for SAT. Sensitivity, specificity, predictive values, and accuracy were computed with their 95% CIs. Agreement between SAT and the reference standard was assessed using Cohen’s kappa coefficient (κ), with values interpreted as: ≤0.20 (slight), 0.21-0.40 (fair), 0.41-0.60 (moderate), 0.61-0.80 (substantial), and 0.81-1.00 (almost perfect). These diagnostic performance metrics were evaluated for the overall cohort and further stratified by predefined clinical and histopathological criteria. All statistical tests were two-sided, and a p-value <0.05 was considered statistically significant. Statistical analyses were performed using SPSS version 12.0 (Chicago, IL, USA).

## Results

### Patient characteristics

**Figure 1:**
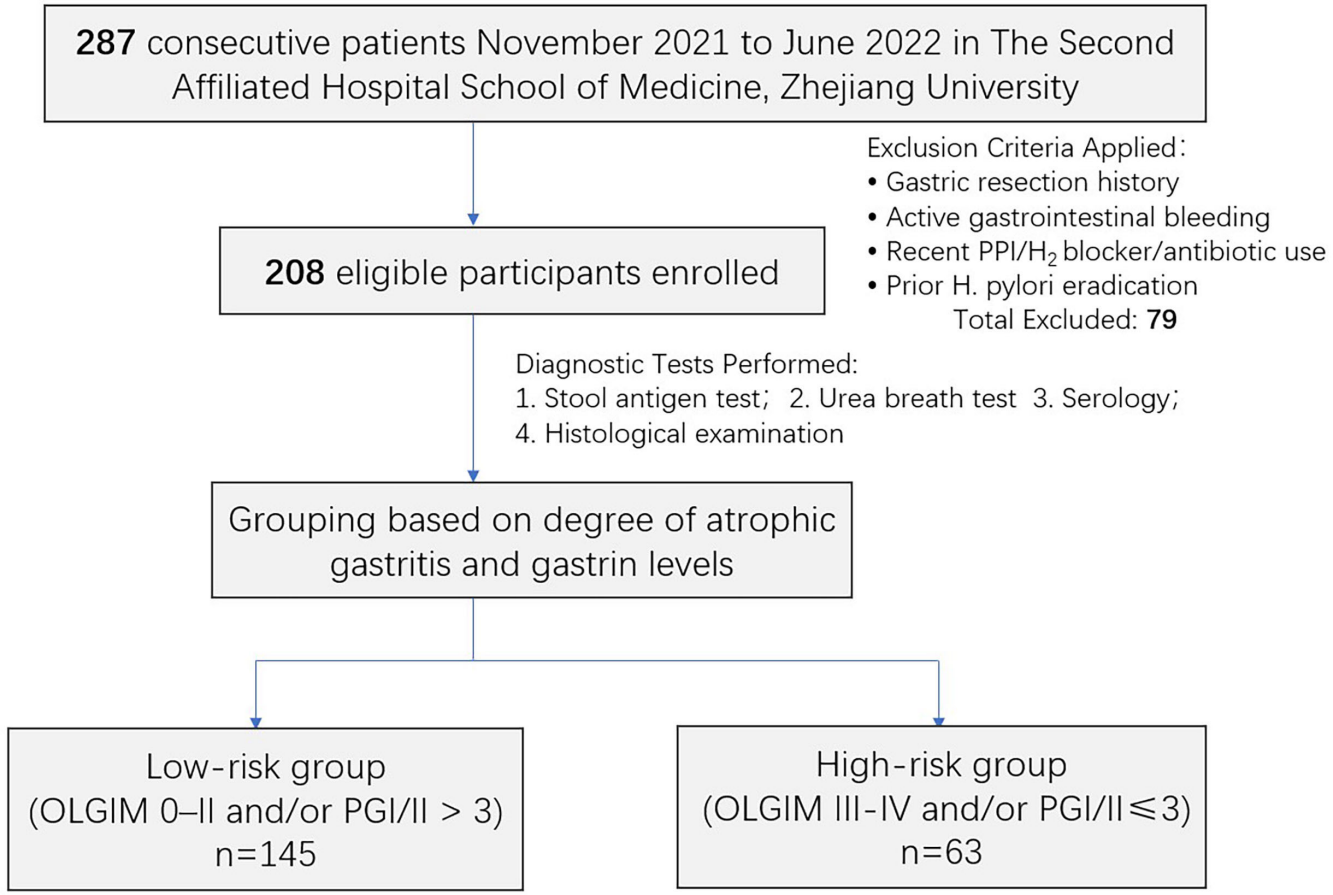
Study enrollment and diagnostic workflow.

In this study, a total of 208 eligible participants (mean age: 52.9 ± 10.2 years) were enrolled (**Figure 1**). The degree and extent of gastric atrophy were evaluated individually for each patient using the Kimura-Takemoto classification, combined with histopathological examination of biopsy specimens. Among the participants, 69.1% (141/204) were diagnosed with mild atrophy accompanied by IM, while 59.9% (124/207) exhibited atrophy confined to a limited area. Based on predefined *H. pylori* status, 100 participants tested positive and 108 tested negative for *H. pylori* infection. Notably, the rate of *H. pylori* positivity was lower in participants with more extensive atrophy (39.7% [27/68]) than in those with limited atrophy (50.3% [70/139]) (see **Table 1**).

**Table 1:** Characteristics of the 208 patients included in the study population.

Characteristics of the 208 patients included in the study population.				
Characteristic	Subgroup	Hp (+)		Hp (-)
		n=100 (%)		n=108 (%)
Gender	Male	48 (48%)		55 (50.9%)
	Female	52 (52%)		53 (49.1%)
Age	≤50	55 (55%)		28 (25.9%)
	> 50	45 (45%)		80 (74.1%)
Severity and extent of gastric atrophy	C0+C1*	65 (65.7%)		59 (55.1%)
	>C2	34 (34.3%)		48 (44.9%)
	Mild	71 (73.9%)		69 (64.5%)
	Moderate/severe	25 (26.1%)		38 (35.5%)
Serum Antibody Test	(+)	73 (85.9%)		9 (9.6%)
	(-)	12 (14.1%)		85 (90.4%)
Stool Antigen Test	(+)	63 (75.9%)		4 (3.9%)
	(-)	20 (24.1%)		98 (96.1%)
Gastric Function Panel	PG-I(≤70)	40 (55.6%)		92 (92.9%)
	PG-I(>70)	32 (45.7%)		7 (7.1%)
	PG-1/PG-II(≤3)	12 (16.2%)		1 (1%)
	PG-1/PG-II(>3)	62 (83.8%)		97 (99%)
	G-17(>15/<1)	2 (2.7%)		5 (5.8%)
	G-17(1-15)	72 (97.3)		81 (94.2%)
Eradicating Hp	Successful	75 (88.2%)		
	Failure	10 (11.8%)		

*Based on the Kimura-Takemoto classification.

### Comparison of diagnostic tests for the detection of *H. pylori* using the predefined reference standard

The sensitivity and specificity of SAT and serum antibody testing were determined based on the combined results of both the urea breath test and histopathological examination as the reference standard. SAT and serum antibody testing demonstrated sensitivities of 75.9% and 85.9%, and specificities of 96.1%, and 90.5%, respectively (see **Table 2**). Although SAT exhibited the highest specificity (96.1%) and positive predictive value (94.0%), its sensitivity (75.9%) was significantly lower than that of serum antibody testing (85.9%, *p* = 0.032, McNemar’s test). This difference in sensitivity was more pronounced in patients with advanced atrophy (C2 stage: SAT 62.1% vs. serum antibody 78.6%, *p* = 0.021). ROC curve analysis revealed that the overall diagnostic accuracy of SAT (AUC = 0.858) was not significantly different from that of serum antibody testing (AUC = 0.882, *p* = 0.18).

**Table 2:** Detection of *H. pylori* using stool antigen test(SAT), and serology.

Performance metric	Stool antigen test	Serum antibody
Sensitivity	75.90%	85.90%
Specificity	96.10%	90.50%
Positive predictive value	94.00%	89.00%
Negative predictive value	83.20%	87.80%
Accuracy	87.03%	88.27%

Sensitivity and specificity of SAT were calculated using a composite reference standard of histology and UBT.

### Optimizing *H. pylori* detection in atrophic gastritis: stratified SAT guided by gastric function markers

Stratified analysis of SAT combined with serum gastric function markers (PG-I, PG-II, and PG-I/PG-II ratio) revealed that in the PG-I abnormal group (≤70 μg/L), specificity (90.5%) and positive predictive value (PPV, 87.5%) were slightly elevated compared to the normal group, while sensitivity remained comparable (see **Table 3**). Of note, a normal PG-I/PG-II ratio (≥3) was associated with improved sensitivity (65.5%) and overall accuracy (78.4%), whereas an abnormal ratio (<3) yielded higher specificity (92.3%), substantially reducing false-positive outcomes. The high specificity observed highlights the potential clinical value of SAT in confirming *H. pylori* eradication after treatment and minimizing unnecessary interventions. In patients with atrophic gastritis (AG), SAT should be prioritized when PG-I/PG-II ratios or PG-I levels are abnormal. A positive result indicates active *H. pylori* infection, while a negative result should be followed by confirmatory testing such as UBT or endoscopy to exclude false negatives.

**Table 3:** Diagnostic Performance of the Stool Antigen Test for H. pylori Stratified by Gastric Function Markers. .

PG-I level	Sensitivity	Specificity	PPV	NPV	Accuracy	n
PG-I (>70)	58.3%	88.9%	77.8%	76.2%	77.1%	42
PG-I (≤70)	63.6%	90.5%	87.5%	70.3%	76.9%	52
PG-I/PG-II ratio						
normal (≥3)	65.5%	88.2%	82.6%	75.0%	78.4%	51
abnormal (<3)	50.0%	92.3%	83.3%	70.6%	72.7%	33
G-17 level						
normal (1-15)	62.1%	90.0%	85.7%	71.4%	76.9%	48
Abnormal (<1 or > 15)	57.1%	83.3%	66.7%	76.9%	72.7%	22

PPV, positive and negative predictive values; NPV, negative predictive values.

### Comparison of *H. pylori* detection results of SAT in patients with different degrees of atrophy

The sensitivity and accuracy of histological diagnosis for detecting AG and/or intestinal metaplasia (IM) have been observed to decrease in individuals with a prior histological diagnosis of these conditions. To better stratify gastric cancer risk, we categorized patients into low-risk (OLGIM stages 0–II and/or PGI/II ratio>3) and high-risk (OLGIM stages III-IV and/or PGI/II ratio ≤ 3) groups based on the severity of AG, IM and serum pepsinogen profiles (Den Hollander et al., 2019). The cohort included 145 patients in the low-risk group and 63 in the high-risk group (see **Table 4**).

**Table 4:** Comparative efficacy of three *H. pylori* diagnostic methods in populations with varying gastric cancer risk. .

Parameter	Low risk group OLGA/OLGIM 0–II and/or PGI/II>3		High risk group OLGA/OLGIM III–IV and/or PGI/II ≤ 3	
	Stool antigen test	Serum antibody	Stool antigen test	Serum antibody
Total positive count	58	59	25	26
Sensitivity	74.14%	84.75%	80.00%	88.46%
Specificity	95.77%	92.19%	96.77%	86.67%
PPV	93.48%	90.91%	95.24%	85.19%
NPV	81.93%	86.76%	85.41%	89.66%
Accuracy	86.05%	88.62%	89.29%	87.50%

PPV, positive and negative predictive values; NPV, negative predictive values.

In the high-risk group, the SAT for *H. pylori* demonstrated comparable specificity (96.77% vs. 95.77%) and PPV (95.24% vs. 93.48%) to those observed in the low-risk group, with numerically higher overall accuracy (89.29% vs. 86.05%). In contrast, serum antibody testing showed slightly reduced performance in high-risk patients, exhibiting lower specificity (81.25% vs. 84.0%), PPV (81.25% vs. 82.86%), and accuracy (83.87% vs. 84.62%) compared to the low-risk group.

### Diagnostic agreement of SAT with the reference standard across risk strata

The agreement between the monoclonal SAT and the predefined reference standard was quantitatively assessed using Cohen’s Kappa statistic. As summarized in **Table 5**, SAT demonstrated substantial agreement with the reference standard in the overall cohort (κ = 0.72, 95% CI: 0.63-0.81). Stratified by gastric cancer risk (Den Hollander et al., 2019), the agreement was notably higher in the high-risk group (κ = 0.77, 95% CI: 0.64-0.90) compared to the low-risk group (κ = 0.69, 95% CI: 0.58–0.80). This indicates that in patients with more advanced atrophy (OLGIM III–IV) and/or abnormal pepsinogen ratios (PG I/II ≤3), the dichotomous outcome of SAT (positive/negative) aligns more closely with the composite diagnostic standard. The superior concordance in the high-risk group, coupled with the test’s high specificity and positive predictive value as previously reported, reinforces the reliability of SAT for confirming active *H. pylori* infection in this clinically critical population.

**Table 5:** Diagnostic performance and consistency of stool antigen test against the reference standard, stratified by gastric cancer risk.

Risk groups	Reference standard positive	Reference standard negative	Total	Consistency metrics
Low-Risk Group (OLGIM 0-II and/or PGI/II >3)				Kappa (κ): 0.69, 95% CI: 0.58 - 0.80;Agreement: Substantial
SAT: Positive	43	3	46	
SAT: Negative	15	84	99	
Subtotal	58	87	145	
High-Risk Group (OLGIM III-IV and/or PGI/II ≤3)				Kappa (κ): 0.77, 95% CI: 0.64 - 0.90, Agreement: Substantial
SAT: Positive	20	1	21	
SAT: Negative	5	30	35	
Subtotal	25	31	56	

CI, Confidence Interval. Agreement level assessed by Landis & Koch criteria.

## Discussion

The diagnostic applicability of the SAT in patients with CAG remains poorly characterized in existing researches. To address this gap, the present study evaluated the performance of this test in a cohort with histologically confirmed CAG. Monoclonal SAT is recognized as a reliable non-invasive method for detecting active *H. pylori* infection, demonstrating high specificity and sensitivity (Korkmaz et al., 2013). It maintains diagnostic efficacy even in the context of advanced AG or IM (Choi et al., 2011). In our cohort of CAG patients, the SAT showed high specificity (96.10%), PPV (94.00%), and overall accuracy (87.03%) in identifying untreated *H. pylori* infection. These results align with previously reported performance metrics for stool-based assays (Archimandritis et al., 1999).

Currently, no single test has been universally established as the gold standard for diagnosing *H. pylori* infection. In prior research, patients with only one positive test result were often classified as indeterminate and consequently excluded from analysis (Blanco et al., 2008). To enhance diagnostic certainty, we therefore required concordance between at least two independent tests. Given the central focus of this study on SAT, stringent criteria for defining positive and negative status were applied: only cases with consistent results from both the UBT and histopathological examination were included. As the UBT is widely accepted as a reference method, its sensitivity, specificity, and accuracy were not re-evaluated in this context (Shin et al., 2009).

CAG is characterized by progressive loss of gastric glandular tissue, often accompanied by intestinal metaplasia (IM), leading to reduced acid secretion and alterations in the gastric microenvironment (Rugge et al., 2002). These pathological changes are known to compromise the diagnostic accuracy of H. pylori tests, particularly biopsy-based methods, due to decreased bacterial colonization density and patchy distribution of infection (Sudraba et al., 2011; Syrjänen et al., 2019).

Consistent with previous reports, we observed that the detection rate of H. pylori by conventional methods declined with advancing atrophy severity. However, the stool antigen test (SAT) demonstrated remarkable stability, with specificity (96.77% vs. 95.77%) and accuracy (89.29% vs. 86.05%) showing no statistically significant differences between risk groups, and even exhibiting a trend toward improved performance in high-risk patients.

Previous studies on SAT performance in patients with gastric atrophy have reported variable results. Charach et al. found a sensitivity of 70% and specificity of 94.4% (Charach et al., 2024), while Choi et al. demonstrated higher sensitivity (93.1%) with comparable specificity (94.6%) and preserved accuracy even in advanced atrophy/intestinal metaplasia (Choi et al., 2011). Our finding that SAT maintains high specificity in high-risk groups with extensive atrophy and IM (96.77%) aligns with the consistently high specificity (94.4-96.1%) reported across these studies. Notably, the sensitivity of SAT in our high-risk group (80.0%) exceeded that reported by Charach et al (Charach et al., 2024). and was also higher than in our low-risk group (74.1%), suggesting that SAT performance may be better preserved in advanced atrophy than previously recognized.

This apparent discrepancy with the conventional understanding that mucosal atrophy invariably diminishes diagnostic performance (Malfertheiner et al., 2017) may be explained by several factors. First, our strict adherence to a 4-week washout period for proton pump inhibitors and antibiotics prior to testing may have reduced false-negative results. Second, the use of a monoclonal antibody-based SAT in our study may offer improved sensitivity compared to polyclonal assays used in earlier investigations. Third, differences in study populations, including the severity and extent of atrophy and intestinal metaplasia, could influence bacterial antigen shedding patterns.

We propose a “biphasic atrophy effect” hypothesis to explain the preserved, and even enhanced performance of SAT in advanced atrophy. In early-stage atrophy (OLGIM I-II), glandular loss reduces the niche for H. pylori colonization, leading to decreased bacterial load and a consequent decline in SAT sensitivity. However, in advanced atrophy with extensive intestinal metaplasia (OLGIM III-IV), the pathophysiological milieu may shift to favor antigen-specific detection. The intestinal metaplastic environment, characterized by reduced acidity (Syrjänen et al., 2019) and altered mucin composition (Arai et al., 2024) could potentially stabilize *H. pylori*-specific antigens during gastrointestinal transit, reducing degradation and enhancing detection. This hypothesis is consistent with the known resistance of secreted mucosal components to proteolytic degradation (Birkholz et al., 1998), and with the established stability of Hp antigens in stool (Vaira et al., 2002). Furthermore, changes in bacterial adherence within metaplastic epithelium, such as upregulation of BabA adhesins in response to altered receptor expression (Ishijima et al., 2011), might modulate antigen shedding in a manner that, while not increasing bacterial load, preserves or enhances immunological detectability. This qualitative shift in antigen release could underlie the superior specificity (96.77%) and PPV (95.24%) observed in our high-risk cohort.

The clinical implications of these findings are substantial. In high-risk patients with advanced atrophy and intestinal metaplasia, SAT’s high specificity (96.77%) and strong agreement with the reference standard (κ = 0.77) position it as a reliable tool for confirming active H. pylori infection. A positive SAT result in this population can guide eradication therapy with high confidence, potentially reducing the need for confirmatory endoscopy. Conversely, given its moderate sensitivity (80.0%), negative results in high-risk patients should be interpreted with caution and may warrant confirmation by UBT to avoid misdiagnosis, particularly important in a population already at elevated gastric cancer risk.

This study has several limitations. First, although SAT proved useful for detecting *H. pylori*, the sensitivity of both SAT and comparative methods remained suboptimal. Second, the use of the UBT as the reference standard precluded direct evaluation of its own sensitivity, specificity, and accuracy relative to other assays. Moreover, the exclusion of patients with only a single positive test, either by UBT or histology, may have introduced selection bias.

In summary, the monoclonal SAT demonstrated diagnostic performance comparable to other established methods for detecting *H. pylori* infection. It remains a clinically useful tool, particularly in patients with CAG or those at high risk for gastric cancer, supporting its rational integration into risk-adapted diagnostic pathways.

## Data Availability

The original contributions presented in the study are included in the article/supplementary material, further inquiries can be directed to the corresponding author/s.
